# Clinical impact of panel gene sequencing on therapy of advanced cancers of the digestive system: a retrospective, single center study

**DOI:** 10.1186/s12885-024-12261-2

**Published:** 2024-04-25

**Authors:** Lena Dreikhausen, Anna Klupsch, Isabella Wiest, Qiyun Xiao, Nadine Schulte, Johannes Betge, Tobias Boch, Christoph Brochhausen, Timo Gaiser, Ralf-Dieter Hofheinz, Matthias Ebert, Tianzuo Zhan

**Affiliations:** 1 Department of Medicine II, Medical Faculty Mannheim, Heidelberg University, Mannheim, Germany; 2 Junior Clinical Cooperation Unit Translational Gastrointestinal Oncology and Preclinical Models, German Cancer Research Center, Heidelberg, Germany; 3 Mannheim Cancer Center, Medical Faculty Mannheim, Heidelberg University, Mannheim, Germany; 4 Department of Medicine III, Medical Faculty Mannheim, Heidelberg University, Mannheim, Germany; 5 Institute of Pathology, Medical Faculty Mannheim, Heidelberg University, Mannheim, Germany; 6 Medical Faculty Mannheim, DKFZ-Hector Cancer Institute, Heidelberg University, Mannheim, Germany

**Keywords:** Targeted therapy, Panel gene sequencing, Cancer therapy, Precision oncology

## Abstract

**Background:**

Panel gene sequencing is an established diagnostic tool for precision oncology of solid tumors, but its utility for the treatment of cancers of the digestive system in clinical routine is less well documented.

**Methods:**

We retrospectively identified patients with advanced or metastatic gastrointestinal, pancreaticobiliary or hepatic cancers who received panel gene sequencing at a tertiary university hospital from 2015 to 2022. For these cases, we determined the spectrum of genetic alterations, clinicopathological parameters and treatment courses. Assessment of actionability of genetic alterations was based on the OncoKB database, cancer-specific ESMO treatment guidelines, and recommendations of the local molecular tumor board.

**Results:**

In total, 155 patients received panel gene sequencing using either the Oncomine Focus (62 cases), Comprehensive (91 cases) or Childhood Cancer Research Assay (2 cases). The mean age of patients was 61 years (range 24–90) and 37% were female. Most patients suffered from either colorectal cancer (53%) or cholangiocellular carcinoma (19%). 327 genetic alterations were discovered in 123 tumor samples, with an average number of 2.1 alterations per tumor. The most frequently altered genes were *TP53, KRAS* and *PIK3CA*. Actionable gene alterations were detected in 13.5–56.8% of tumors, according to ESMO guidelines or the OncoKB database, respectively. Thirteen patients were treated with targeted therapies based on identified molecular alterations, with a median progression-free survival of 8.8 months.

**Conclusions:**

Actionable genetic alterations are frequently detected by panel gene sequencing in patients with advanced cancers of the digestive tract, providing clinical benefit in selected cases. However, for the majority of identified actionable alterations, sufficient clinical evidence for targeted treatments is still lacking.

**Supplementary Information:**

The online version contains supplementary material available at 10.1186/s12885-024-12261-2.

## Introduction

Cancers of the digestive system represent a major fraction of the global tumor burden [1, 2]. Despite their high incidence, the therapeutic options for most advanced and metastatic cancers of the digestive system are still limited. Molecular profiling approaches, in particular next-generation tumor genome sequencing, hold the promise of identifying alterations that can be exploited for cancer therapy. This concept of personalized oncology is supported by several large prospective clinical trials showing that actionable mutations can be detected in in 36.7–58.2% of patients with solid cancers [3, 4]. Furthermore, results of these studies indicate that genomics-driven cancer therapy can improve overall survival and reduce toxicity-related mortality [5]. Hence, precision oncology approaches have been integrated into routine oncological care in many academic medical centers. Retrospective real-world data from these centers suggest that actionable mutations can be detected in 20–40% of cancer patients [6, 7], matching findings from prospective studies [3, 4]. For cancers of the digestive systems, studies have reported that actionable genetic alterations can be found in 5.8 to 27.8% of cases, with highest rates in cholangiocellular and gastroesophageal cancers, and lowest rates in pancreatic cancer [8]. Different methods for high-throughput DNA profiling have been applied for precision oncology, ranging from whole genome/exome sequencing to more focused approaches such as panel gene sequencing. While mutational profiling by whole genome/exome sequencing is comprehensive, it is also associated with higher costs and requires more extensive bioinformatic resources for analysis compared to more focused sequencing approaches [9]. In contrast, gene panel sequencing is versatile and less costly, but allows only the assessment of hotspot mutational sites in predefined sets of genes [9]. In clinical practice, translation of cancer genomics into novel therapeutic options for patients is frequently hindered by a lack of access to suitable clinical trials, delayed coverage of therapy cost by insurance companies or rapid disease progression [10]. Thus, many studies indicate that only a minor fraction of patients with actionable genetic alteration benefit from alteration-specific therapies [6, 11]. While the real-life clinical utility of panel gene sequencing has been evaluated across a broad spectrum of solid tumors [12, 13], few studies focused on cancers of the digestive system.

In this retrospective study, we determined the results of panel gene sequencing in 155 patients with locally advanced or metastatic cancers of the digestive system who were treated at a tertiary academic medical center. We describe the spectrum of identified mutations, subsequent mutation-specific therapeutic approaches, and clinical courses of the patients receiving tailored therapies.

## Methods

### Collection of clinical data

Patient selection was performed by a database query of the local data integration center of the Mannheim University Hospital. All histopathological reports generated by the Institute of Pathology of Mannheim University Hospital between 01/2015 and 03/2022 were searched for documentation of the term “oncomine”, which comprises the following panel gene sequencing assays: Oncomine Focus Assay, Oncomine Comprehensive Assay and Oncomine Childhood Cancer Research Assay. Among all identified patients, those who were >18 years and encoded with the following ICD codes were selected for analysis: malignant neoplasm of the esophagus (C15), stomach (C16), small intestine (C17), colon (C18), rectosigmoid junction (C19), rectum (C20), anus and anal canal (C21), liver and intrahepatic bile ducts (C22), gallbladder (C23), biliary tract (C24), pancreas (C25) and other imprecisely defined digestive organs (C26). Next, only patients with locally advanced or metastatic cancers who required systemic therapies were included for in-detail review. A detailed list of all included cancer entities can be found in Supplementary Table 1. For this patient cohort, we obtained information on demographic parameters (age, sex), cancer entity and stage, lines of systemic therapy and progression-free survival (PFS). PFS was defined as time from initiation of molecular-targeted therapy until radiological disease progression or time of data cut-off (31.12.2022).

### Collection of panel sequencing and pathology data

From the panel gene sequencing reports, we obtained the following data: type of assay, number, and types of identified genetic alterations, allele frequency and assessment of pathogenicity. Three panel sequencing assay types were applied in our cohort: the Oncomine Focus panel covers 52, the Comprehensive Panel v3 161 and Childhood Cancer Research Panel 203 unique cancer-related genes. Assay types and versions did not change during the observation period. A list of individual genes that are covered by the respective assays can be found in Supplementary Table 2. For sequencing, fresh-frozen paraffin-embedded (FFPE) tissue samples were used in all cases. The minimum input of genomic DNA was 10 ng and sequencing was performed using a combination of Thermo Fisher Ion GeneStudio S5 and Ion Chef System.

Furthermore, we collected information on complementary immunohistochemical markers including Her2 amplification and deficient mismatch repair (dMMR) from routine pathology reports. dMMR was determined by immunohistochemical staining of MLH1, PMS2, MSH2 and MSH6. Tumors with loss of expression of at least one of these proteins were considered as dMMR.

### Assessment of actionability of genetic alterations

Actionability of genetic alterations was retrospectively assessed on a tumor entity level using the OncoKB database [14] and the ESMO guidelines for the management of colorectal, gastric, esophageal, pancreatic, biliary and hepatocellular cancers [15–20]. Clinical evidence supporting actionability of mutations was categorized using the OncoKB score or the ESMO-ESCAT score [21] as proposed in the ESMO guidelines. Furthermore, we included recommendations of the local molecular tumor board if available. Assessment of actionability by the molecular tumor board was based on the NCT/DKTK classification [22]. A detailed explanation of the scoring system of the three classifiers can be found in Supplementary Table 3.

### Statistical analysis

The Wilcoxon-rank sum test was used to compare number of detected mutations between different panel sequencing assay types. All figures were generated using Graph Pad Prism, version 9.

## Results

### Clinico-pathological characteristics of cancer patients

**Fig. 1:**
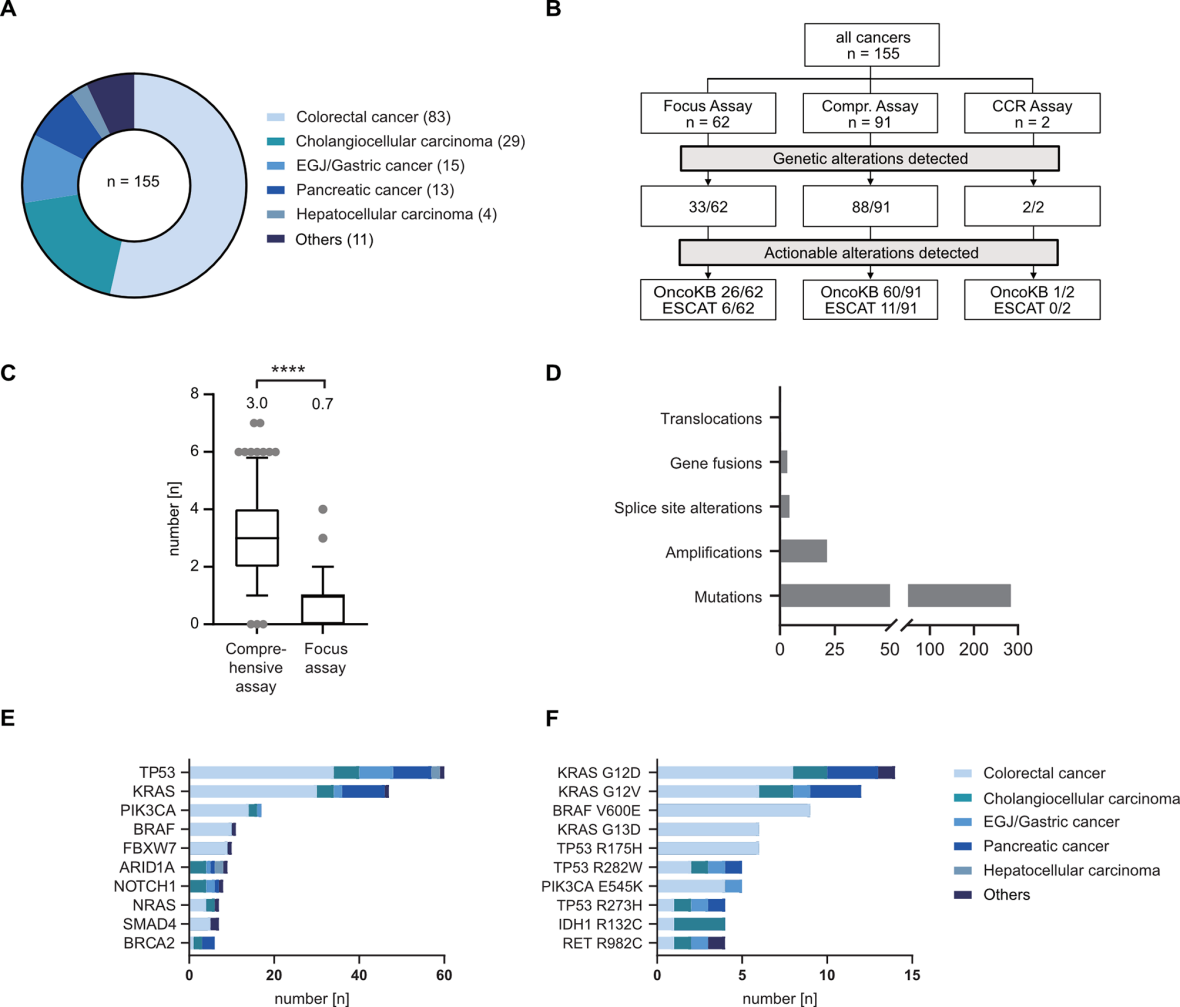
Spectrum of cancer mutations detected by panel gene sequencing. (**A**) Pie chart showing distribution of cancer entities in panel gene sequencing cohort. (**B**) Flowchart showing distribution of assay types, and the fraction of assays that detected genetic alterations or actionable alterations. Actionability of genetic alterations were either classified using the OncoKB database (“OncoKB”) or the ESMO guidelines (“ESCAT”). (**C**) Frequency of detected mutations between assay types are shown as box plots with whiskers indicating 10/90 percentiles. The mean number of genetic alterations per assay is shown above the box plots. Statistical testing was done using a Wilcoxon-rank sum test. **** indicates *p* < 0.001. (**D**) Bar chart showing distribution of different genetic alterations in the cohort. (**E-F**) Bar chart showing the ten most frequently altered genes (**E**) and the most frequent genetic alterations (**F**). *Abbreviations*: CCR– Childhood Cancer Research (Assay)

We retrospectively identified a total of 155 patients who were treated for locally advanced or metastatic cancers of the digestive system and received in-house panel gene sequencing. Median age of this cohort was 61 years (range 24–90 years) and 98 (63%) patients were male. The most frequent cancer entities were colorectal cancers (CRC, 83 cases), followed by cholangiocellular carcinomas (CCC, 29 cases) and esophageal/esophagogastric junction/gastric cancers (15 cases) (Fig. 1A). Rare cancer types in this cohort included one case of duodenal adenocarcinoma, one of neuroendocrine carcinoma of the esophagogastric junction, one of neuroendocrine tumor of the midgut, one of small bowel adenocarcinoma, two of ampullary cancers, two of mixed hepatocellular-cholangiocellular carcinomas and three of cancers of unknown primary (total 11 cases).

Median time from first diagnosis of the disease to panel gene sequencing was 23.4 months, with the longest median time periods for patients with hepatocellular carcinoma (34.8 months). For panel sequencing, biopsy material from either metastatic lesions (68 cases) or primary tumors (86 cases) was used. In one case, biopsy from a local tumor recurrence was analyzed. The majority of biopsies was obtained during diagnosis of the disease. For most cancer types, panel gene sequencing was performed during first or second-line therapy, whereas it was performed during third-line therapy in patients with hepatocellular carcinoma (HCC).

The most frequently applied panel gene sequencing assay was Oncomine Comprehensive Assay v3 (91), followed by Oncomine Focus Assay (62) and Oncomine Childhood Cancer Research Assay (2) (Fig. 1B). Selection of assay type was based on individual oncologists’ preferences. Distribution of assay types was not balanced between tumor entities. The Oncomine Focus Panel was most frequently used in CRC (40/83) while the Oncomine Comprehensive Panel was mostly applied in cholangiocellular carcinoma (16/29), esophagogastric junction cancer (9/15), pancreatic adenocarcinoma (12/13), and hepatocellular carcinoma (4/4).

Data on complementary predictive immunohistochemical markers such as Her2 amplification and microsatellite instability were collected from routine pathology reports. Her2 expression status was available from 55 patients and was negative in all cases. Mismatch repair deficiency was assessed in 128 cases and could be detected in tumors of five patients with CRC, one with CCC and one with duodenal adenocarcinoma. Clinico-pathological characteristics are displayed in Table 1.

**Table 1:** Distribution of age, gender and positivity for immunohistochemical biomarkers

	All (*n* = 155)	CRC (*n* = 83)	CCC (*n* = 29)	EGJ (*n* = 15)	PDAC (*n* = 13)	HCC (*n* = 4)	Others (*n* = 11)
Age [yrs]							
Median (Range)	61 (24–90)	60 (25–90)	63 (38–78)	58 (24–84)	62 (39–82)	58 (49–64)	63 (32–81)
Gender, n [%]							
Male	98 [63%]	45 [54%]	20 [69%]	13 [87%]	7 [54%]	4 [100%]	9 [82%]
Female	57 [37%]	38 [46%]	9 [31%]	2 [13%]	6 [46%]	0	2 [18%]
Mismatch repair status, n [%]							
pMMR	121 [78%]	74 [89%]	21 [73%]	13 [87%]	7 [54%]	0	6 [55%]
dMMR	7 [5%]	5 [6%]	1 [3%]	0	0	0	1 [9%]
ND	27 [17%]	4 [5%]	7 [24%]	2 [13%]	6 [46%]	4 [100%]	4 [36%]
Her2-status, n [%]							
Her2-negative	55 [36%]	22 [27%]	13 [45%]	13 [87%]	3 [23%]	0	4 [36%]
Her2-positive	0	0	0	0	0	0	0
ND	100 [64%]	61 [73%]	16 [55%]	2 [13%]	10 [77%]	4 [100%]	7 [64%]

*Abbreviations: CRC* Colorectal cancer, *CCC* Cholangiocellular carcinoma, *EGJ* Esophagogastric junction adenocarcinoma, *PDAC* pancreatic ductal adenocarcinoma, *HCC* Hepatocellular carcinoma, *pMMR* proficient mismatch repair, *dMMR* deficient mismatch repair, *ND* Not determined

### Spectrum of genetic alterations determined by panel gene sequencing

Next, we determined the spectrum of genetic alterations that was detected by panel gene sequencing. On average, 2.1 alterations were detected per tumor sample (range 0–7). No genetic alterations were detected in 32 cases, of which 29 used the Oncomine Focus panel (Fig. 1B). We observed a significant difference in median number of detected alterations between assay types (Oncomine Focus Assay: 0.7 per sample, Oncomine Comprehensive Assay: 3.0 per sample, Fig. 1C). Between tumor entities, the mean number of detected alterations also differed (e.g. 1.8 alterations per sample in CCC versus 3.1 alterations in pancreatic adenocarcinoma). In total, 327 genetic alterations were detected, of which 191 were classified as pathogenic or likely pathogenic by the Oncomine database. Most genetic alterations were gene mutations (89.3% of all alterations), while copy number changes (7%), gene fusions (1.5%), splice site alterations (1.8%) and translocations (0.3%) were detected in a minority of cases (Fig. 1D). Mutations were predominantly detected in *TP53* (61), *KRAS* (47), *PIK3CA* (17), *BRAF* (11) and *FBXW7* (10). The most common single mutations were *KRAS G12D* (14), *KRAS G12V* (12), *BRAF V600E* (9) and *KRAS G13D* (6), predominantly detected in CRC and pancreaticobiliary cancers (Fig. 1E and F). Average allele frequency of detected mutations was 38% (range: 3.85–100%). Twenty-three copy number changes were detected in 4 cancer entities (CRC, hepatocellular carcinoma, esophagogastric cancer and pancreatic adenocarcinoma). The most common copy number changes were observed in *MYC* (3) and *EGFR* (2). Gene fusions detected include *PTPRK-RSPO3* (3), *GOPC-ROS1* (1) and *BRD4-PPARG* (1), all observed in CRC. Splice site alterations were found in *RICTOR* in hepatocellular carcinoma and ampullary cancer (2), as well as in *CDKN2A* in CCC (1) and in *PMS2* (1), *TP53* (1) and *FBXW7* (1) in CRC.

### Actionability of genetic alterations detected by panel gene sequencing

We then determined the proportion of genetic alterations for which genomics-directed therapies have been described, by using different resources. First, we used the publicly accessible OncoKB precision oncology database [14]. Here, actionable alterations are assigned to four levels of evidence, based on published clinical and preclinical studies (see Supplementary Table 3 for explanation of scores). Using the OncoKB database, 35.8% (117/327) of all identified alterations were classified as actionable. Classifications of actionable alterations were as follows: 10% were assigned to level 1, 2% to level 3A, 38% to level 3B and 50% to level 4. We observed a difference in the frequency of detected, actionable alterations between assay types, with 66% of all Oncomine Comprehensive Assays and 42% of all Oncomine Focused Assays detecting at least one actionable mutation.

**Fig. 2:**
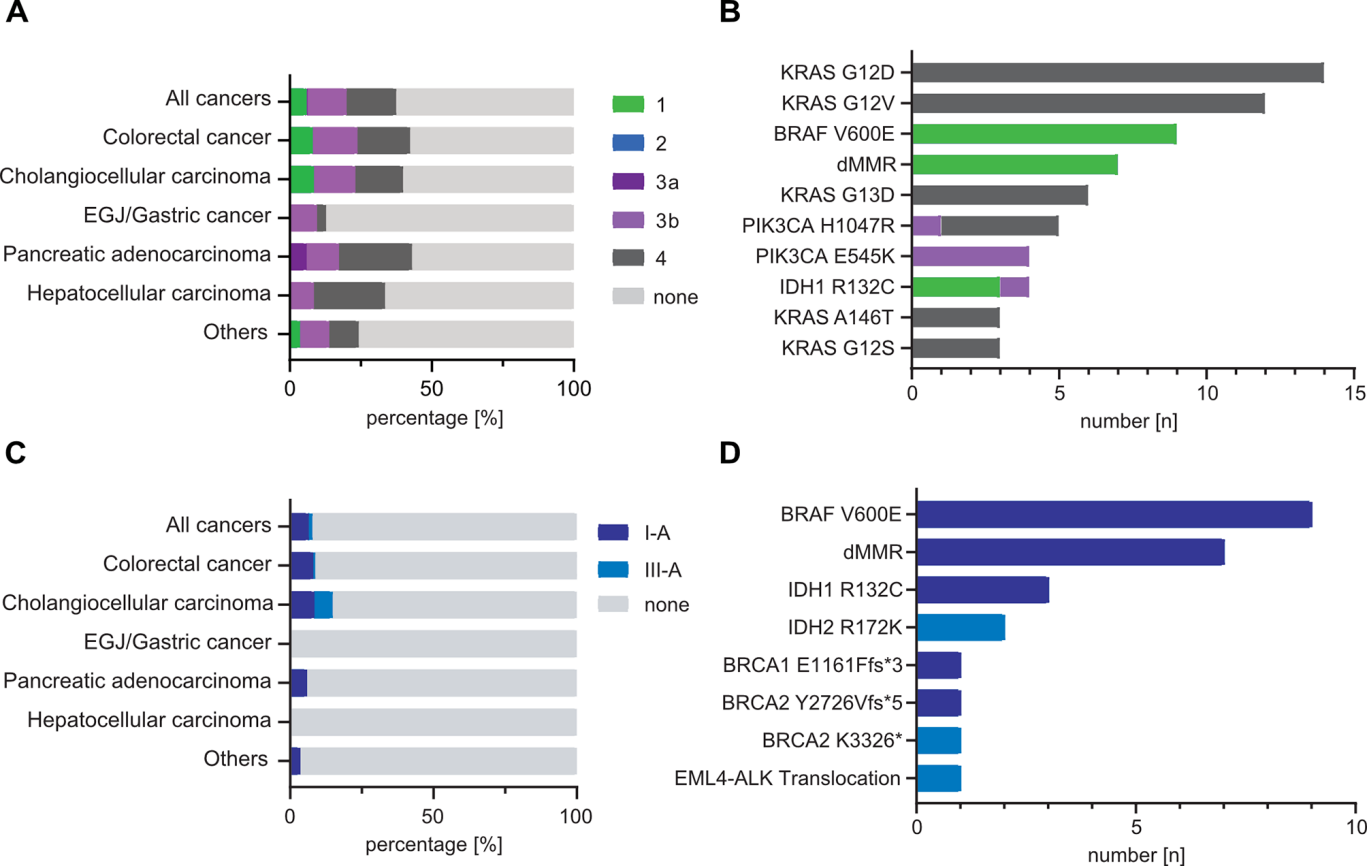
Spectrum of detected actionable genetic alterations. (**A**-**B**) Bar chart showing the fraction of actionable mutations per cancer entity (**A**) and ten most frequent actionable genetic alterations (**B**), as assessed and classified using the OncoKB database. (**C**-**D**) Bar chart showing the fraction of actionable mutations per cancer entity (**C**) and all actionable genetic alterations (**D**), as assessed by the EMSO treatment guidelines for gastrointestinal, hepato- and pancreaticobiliary cancers and classified by the ESCAT score

Next, we evaluated actionability of genetic alterations using the ESMO guidelines for gastrointestinal, hepato- and pancreaticobiliary cancers [15–20]. In the ESMO guidelines, the ESMO-ESCAT classification was used to assess the clinical evidence underlying actionable genetic alterations [21] (see Supplementary Table 3). In total, only 5.5% (18/327) of alterations were considered as actionable by the ESMO guidelines, with 14 classified as I-A and 4 as III-A by the ESMO-ESCAT score. Except for the BRCA K3326* mutation, all other alterations were also classified as actionable by OncoKB. Deficient mismatch repair (dMMR) is considered as an actionable alteration with high level of evidence by both the OncoKB database and ESMO guidelines. After including 7 cases with dMMR, actionable alterations were found in 56.8% (88/155) and 13.5% (21/155) of tumors/patients, using OncoKB and ESMO guidelines respectively (Fig. 2A-B). Actionable alterations with highest levels of evidence (OncoKB level 1 or ESMO-ESCAT I-A) occurred most frequently in CRC and CCC, and included dMMR, *BRAF V600E*, and *IDH R132C* (Fig. 2C and D). In many tumors, multiple actionable alterations were detected (most frequently *KRAS* plus *PIK3CA* mutations or dMMR plus *BRAF V600E* in CRC). Only one druggable gene translocation was identified in our cohort (ELM4-ALK2 in colorectal cancer, ESMO-ESCAT level III-A).

We also reviewed recommendations by the local molecular tumor board which was introduced at our university hospital in 2020. A total of 31 cases from our cohort were presented in the molecular tumor board and actionable alterations could be identified in 17 tumors/patients. In accordance with national guidelines [23], the most frequently presented tumor entity in the molecular tumor board was CCC. Actionability of mutations was most frequently identified for the following genes: *ARID1A*, *ERBB3, SMARCA4* and *PIK3CA*. A list of all identified actionable alterations and their recommended drugs is presented in Supplementary Table 4.

### Clinical courses of cancer patients with actionable alterations

A total of 13 patients received one or more tailored therapies, including seven patients with CRC, four with CCC, one with duodenal adenocarcinoma and one with pancreatic adenocarcinoma (Table 2). Except for two cases, all actionable alterations belonged to the highest evidence levels (OncoKB evidence level 1, ESMO-ESCAT level I-A). The median number of preceding lines of therapy in this cohort was 1 (range 0–4). The most frequent alterations were dMMR (6), treated with pembrolizumab or nivolumab/ipilimumab, and *BRAF V600E* (4), treated with encorafenib/cetuximab +/- binimetinib. We also found two patient cases of *IDH1 R132C* mutations in CCC, who were treated with the IDH1 inhibitor ivosidenib. At data cut-off (December 2022), median PFS of this patient cohort was 8.8 months (range 2.3–67.3 months) and in 6 patients, biomarker-directed therapy was ongoing. Best responses to therapy were one case with complete response, four with partial response, three with mixed response, four with stable disease and one with progressive disease. PFS was longest in patients with dMMR CRC receiving pembrolizumab (median 12.4 months, four patients) and shortest in patients with *IDH1 R132C* mutant CCC who were treated with ivosidenib (median 2.4 months, two patients).

**Table 2:** Clinical courses of patients receiving genetic alteration-driven therapies. PFS for molecular-directed therapy is calculated as months until data cut-off (31.12.2022)

Cancer type, tumor stage at time of molecular-directed therapy	Genetic alteration	Drug	Lines of pretreatments (n)	Time on prior therapy (months)	PFS for molecular-directed therapy (months)	Best response
CCC, UICC IV	*IDH1* *R132C*	Ivosidenib	1	12.4	2.5	progress
CCC, UICC IV	*IDH1* *R132C*	Ivosidenib	3	11.4	2.3	stable disease
CCC, UICC IV	*ERBB3* *G284R*	Lapatinib + Trastuzumab	2	10.3	7.3 (ongoing)	mixed response
CCC, UICC IV	dMMR	Pembrolizumab	1	3.7	65.1 (ongoing)	complete response
CRC, UICC IV	dMMR/ *BRAF* *V600E*	Pembrolizumab/ Encorafenib + Cetuximab	0	0	12.2 (9.2/3.0)	stable disease/ progress
CRC, UICC IV	dMMR/ *BRAF V600E*	Pembrolizumab	0	0	15.5 (ongoing)	stable disease
CRC, UICC IV	dMMR	Pembrolizumab/ Nivolumab + Ipilimumab	0	0	28.8 (27.6/1.2)	partial response/ n.a.
CRC, UICC IV	*BRAF V600E*	Encorafenib + Binimetinib + Cetuximab	1	1.4	5.1	partial response
CRC, UICC IV	dMMR	Pembrolizumab	0	0	8.9	mixed response
CRC, UICC IV	*BRAF V600E*	Encorafenib + Cetuximab	0	0	3.8	mixed response
CRC, UICC IV	*BRAF V600E*	Encorafenib + Cetuximab	0	0	0.8 (ongoing)	Not assessed (ongoing)
Duodenal adenocarcinoma, UICC IV	dMMR	Pembrolizumab/ Nivolumab	1	3	39.6 (36.3/3.3)	stable disease
PDAC, UICC IV	BRCA1 E1161Ffs*3/ *BRCA2 S497L*	Olaparib	3	19.3	9.0 (ongoing)	Partial response

*CCC* Cholangiocellular carcinoma, *CRC* colorectal cancer, *PDAC* pancreatic ductal adenocarcinoma, *dMMR* deficient mismatch repair, *ND* not discussed (cases were not presented in local molecular tumor board), *UICC* Union for International Cancer Control tumor stage

Two patients received therapies following the recommendations of the local molecular tumor board, as mutations with only low or no levels of evidence for actionability were detected (*BRCA2 S497L* in pancreatic adenocarcinoma; *ERBB3 G284R* in CCC). The patient with pancreatic adenocarcinoma presented with a germline loss-of-function mutation of *BRCA1* (E1161Ffs*3) and germline mutation of unknown significance in *BRCA2* (*S497L*). Two months after completion of neoadjuvant chemotherapy with eight cycles of FOLFIRINOX, pancreaticoduodenectomy was performed. Subsequently, the patient developed recurrent and metastatic disease which progressed under six cycles of treatment with gemcitabine/nab-paclitaxel. Therapy was switched to 5-fluorouracil/folic acid/nanoliposomal irinotecan but had to be discontinued due to uncontrollable diarrhoea and fatigue, despite radiological partial disease response. Due to severe neuropathy, re-induction with platin-based chemotherapy regimens was not possible. He then received olaparib monotherapy based on recommendations of the molecular tumor board, with an ongoing treatment response of nine months at data cut-off. The patient with CCC and *ERBB3 G284R* mutation received a combination therapy with lapatinib and trastuzumab after disease progression under treatment with gemcitabine/cisplatin, with an ongoing disease control for 7.3 months.

## Discussion

This retrospective, real-world analysis of 155 patients with advanced cancers of the digestive system shows that gene panel sequencing can uncover actionable genetic alterations that result in individualized therapeutic options.

In our patient cohort, actionable molecular alterations were observed in 13.5–56.8% of cases, using the ESMO treatment guidelines or OncoKB database, respectively. When using OncoKB as reference, our number of detected actionable alterations is comparable to published, prospective studies that used panel gene sequencing and reported frequencies between 36.7 and 58.2% [3, 4]. However, significantly fewer actionable alterations were detected when the ESMO treatment guidelines were applied.

In general, our study shows that the frequency of identified actionable alterations was dependent on two main factors: the genomic coverage of the assay type and the classifier used. In 29 out of 32 cases in which no mutations could be detected, a focused panel gene sequencing assay was used. Similarly, the percentage of tumors with actionable mutations, as classified by the OncoKB database, was significantly lower when the Oncomine Focused assay was used (Fig. 2B). These findings indicate that to maximize the clinical utility of gene panel sequencing, comprehensive gene panels are needed.

Secondly, we observed that the number of actionable alterations significantly differed between the OncoKB database and ESMO treatment guidelines. We found a major overlap between the two resources for actionable alterations with high levels of clinical evidence, such dMMR or *BRAF V600E*. However, the OncoKB database also includes many actionable genetic alterations with low levels of clinical evidence which were not evaluated by the ESMO guidelines and thus not assigned an ESMO-ESCAT score. This difference in classification is most apparent for alterations in *KRAS*, which was the second most frequently mutated gene in our cohort. While OncoKB suggested MEK inhibitors as a treatment for *KRAS* mutant cancers, most early clinical trials did not find any clinical benefit of MEK inhibitor monotherapy in gastrointestinal cancers [24]. In contrast, only *KRAS G12C* was classified as an actionable target by the ESMO guidelines, which is supported by several clinical trials [25–27]. Overall, *KRAS G12C* mutations are rare in cancers of the digestive tract and we only detected one case in a patient with pancreatic cancer. For this patient, sotorasib was recommended by the molecular tumor board, but treatment with olaparib was initiated due to concurrent *BRCA1/2* mutations. However, as other genotype-specific or pan-KRAS inhibitors are currently undergoing (pre-)clinical testing [28], the importance of *KRAS* mutations as an actionable target will steadily increase.

In general, it is challenging to compare the frequencies of detected actionable alterations between panel gene sequencing studies, as the actionability can be assessed by many different approaches. Besides the OncoKB database, several other public databases (e.g. Clinical Interpretation of Variants in Cancer [29], Cancer Genome Interpreter [30]), and commercial resources (e.g. Jackson Laboratory Clinical Knowledge Base [31]) exist that assess actionability of genetic alterations. Depending on the evidencelevels that are defined as thresholds by these databases and the depth of literature research, the actionability of genetic alterations might be assessed differently. Furthermore, in many past studies, treatment selection was guided by the recommendations of the local molecular tumor boards or clinical experts [32, 33], providing another layer of heterogeneity. To reduce this heterogeneity of recommendations, an important step would be to establish publicly accessible, transparent databases that collect clinical and pre-clinical evidence for molecular-directed therapies. These databases would enable a stronger unification of treatment recommendations, especially for druggable mutations that occur at low frequencies and for which clinical evidence is rare. An ongoing data curation would be necessary as well as regular validation of recommendations by expert panels.

Lastly, the frequency of actionable alterations also depends on the composition of tumor entities in the respective cohorts. Several recent retrospective studies have shown that panel sequencing can uncover actionable mutations across different solid tumors at high rates [12, 13]. However, cancers of the digestive system only account for a small fraction in these studies and the frequency of actionable alterations is less clear in this subgroup. The large proportion of CRC and CCC in our cohort might introduce a bias towards cancer types of the digestive system that have a comparatively high frequency of actionable mutations.

Due to its retrospective design, our study has some limitations. First, we included three panel gene sequencing assay types which differed in their coverage of genetic alterations. We also observed differences in the usage of these assays depending on the tumor entity. The selection of the assay type was not based on predefined algorithms but rather on individual clinical decisions. Thus, comparing frequencies of specific mutations between tumor types must be regarded with care, as the data might be biased by differential selection of panel sequencing assays. To overcome this problem in clinical practice, guidelines that clearly define the optimal time point and assay type for specific tumor entities should be implemented. Secondly, performance of panel gene sequencing assays depends on the quality of the tissue material [34]. In some cases, FFPE tissue from primary cancers were used that have been stored over longer time periods. This might affect the quality of DNA and specifically of RNA, leading to lower sensitivity for specific alterations such as gene fusions [35]. Lastly, our data indicates that those patients who received a genomics-directed therapy had an overall high clinical benefit. However, this observation is biased by the large proportion of patients with dMMR CRC who received immune checkpoint inhibitors which are highly effective in this tumor subtype [36]. In contrast, the clinical benefit of ivosidenib in CCC (median PFS 2.4 months in two patients) or cetuximab/encorafenib in CRC (median PFS 3.4 months in 4 four patients) was less pronounced, but matching results of the respective trials (median PFS of 2.7 months for ivosidenib [37]and median PFS 4.2 for cetuximab/encorafenib [38].

Despite these limitations, our study shows that selected patients can benefit greatly from molecular-directed therapies. We report two cases of mutations with low evidence for actionability based on the OncoKB database (*BRCA2 S497L* and *ERBB3 G284R*), but for which our local molecular tumor board suggested therapeutic options that led to sustained tumor responses. Based on the results of the POLO trial, patients with pancreatic carcinoma with germline loss-of-function *BRCA2* mutations benefit from olaparib maintenance therapy following disease control with first-line oxaliplatin-based chemotherapy regimen [39, 40]. While the trial only included deleterious and likely deleterious germline mutations, the clinical impact of non-synonymous germline mutations such as *BRCA2 S497L* remain unknown [41]. Here, we report the case of a patient with metastatic pancreatic carcinoma with a combination of germline BRCA1 E1161Ffs*3 and *BRCA2 S497L* mutations that benefit from treatment with olaparib monotherapy after failure of several lines of preceding systemic therapies. Missense mutations of *BRCA2* can moderately increase the risk for breast cancer [42], but it is unknown if they also modulate the response to PARP inhibitors. Considering the durable response that we observed in our patient case, further preclinical studies should be performed to decipher the actionability of *BRCA2* missense mutations. *ERBB3* mutations can occur in various cancer entities, but are overall rare events [43]. Mutations of *ERBB3* can induce activation of the MAPK pathway, and a previous case report showed that targeting *ERBB3 G284R* mutant breast cancer with trastuzumab plus lapatinib can lead to disease control [44]. The *ERBB3 G284R* mutation is considered an activating alteration [45], and to our knowledge, our case shows for the first time that treatment with trastuzumab/lapatinib can result in prolonged disease control in metastatic CCC with this specific *ERBB3* mutation.

## Conclusions

In summary, our study shows that panel gene sequencing can reveal actionable genetic alterations in cancers of the digestive tract, with clinical benefit for selected patients in real-life. The utility of panel gene sequencing depends on the genomic coverage of the panel and comprehensive gene panels should be used. In-depth assessment of identified mutations by molecular tumor boards can identify novel actionable genetic alterations that are not enlisted in public precision medicine databases.

### Electronic supplementary material

Below is the link to the electronic supplementary material.

Supplementary Material 1

## Data Availability

No datasets were generated or analysed during the current study.

## Abbreviations

CCC: Cholangiocellular carcinoma
CRC: Colorectal cancer
ESCAT: ESMO Scale for Clinical Actionability of Molecular Targets
ESMO: European Society For Medical Oncology
FFPE: Fresh-frozen paraffin-embedded
HCC: Hepatocellular carcinoma
NCT/DKTK: National Center for Tumor Diseases/Deutsches Konsortium für Translationale Krebsforschung (German Cancer Consortium)
dMMR: Mismatch repair deficiency
PFS: Progression-free Survival
