# Supplementary material for: Clinical impact of panel gene sequencing on therapy of advanced cancers of the digestive system: a retrospective, single center study
Source: BMC Cancer. 2024 Apr 25;24:526. doi: 10.1186/s12885-024-12261-2 (PMC11046933; doi:10.1186/s12885-024-12261-2)
Supplement: Supplementary file 1 — Supplementary Material 1 [file 12885_2024_12261_MOESM1_ESM.docx]

**Additional File 1:**

**Supplementary Table 1: Distribution of tumor entities.**

| **Tumor type** | **Number of patients** |
| --- | --- |
| Esophagogastric junction/Gastric cancer | 15 |
| Cholangiocarcinoma | 29 |
| Colorectal cancer | 83 |
| Pancreatic adenocarcinoma | 13 |
| Hepatocellular carcinoma | 4 |
| Others | 11 |
| - Small bowel adenocarcinoma | 2 |
| - Neuroendocrine carcinoma of the esophagogastric junction | 1 |
| - Neuroendocrine tumor of the midgut | 1 |
| - Ampullary cancer | 2 |
| - Mixed hepatocellular-cholangiocellular carcinoma | 2 |
| - Cancer of unknown primary | 3 |
| **All cancers** | **155** |

**Supplementary Table 2: Characteristics of panel sequencing assay types.**

| **Oncomine Focus Assay** |
| --- |
| ***- Hotspot Genes (Exons) (n=35); DNA***  AKT1(1,3), ALK(21-25), AR(6,8), BRAF(11,15), CDK4(2), CTNNB1(3), DDR2(5), EGFR(3,7,12,15,18,19,20,21), ERBB2(8,17-22), ERBB3(2,3,6,8,9), ERBB4(18), ESR1(9), FGFR2(7,8,9,12,14), FGFR3(7,9,14,16), GNA11(4,5), GNAQ(4,5), HRAS(2,3), IDH1(4), IDH2(4), JAK1(14,15,16), JAK2(14), JAK3(11,12,15), KIT(8,9,11,13,17), KRAS(2,3,4), MAP2K1(2,3,6), MAP2K2(2), MET(14,16,19), MTOR(30,39,40,43,47,53), NRAS(2,3,4), PDGFRA(12,14,18), PIK3CA(2,5,6,8,10,14,19,21), RAF1(7,12), RET(10,11,13,15,16), ROS1(36,38), SMO(4,6,8,9)  ***- Copy Number Variations (n=19); DNA***  ALK, AR, BRAF, CCND,- CDK4, CDK6, EGFR, ERBB2, FGFR1, FGFR2, FGFR3, FGFR4, KIT, KRAS, MET, MYC, MYCN, PDGFRA, PIK3CA  ***- Fusion Drivers (n=23); RNA***  ABL1, AKT3, ALK, AXL, BRAF, EGFR, ERBB2, ERG, ETV1, ETV4, ETV5, FGFR1, FGFR2, FGFR3, MET, NTRK1, NTRK2, NTRK3, PDGFRA, PPARG, RAF1, RET, ROS1 |
| **Oncomine Comprehensive Assay v3** |
| ***- Completely Covered Genes (n=48); DNA***  ARID1A, ATM, ATR, ATRX, BAP1, BRCA1, BRCA2, CDK12, CDKN1B, CDKN2A, CDKN2B, CHEK1, CREBBP, FANCA, FANCD2, FANCI, FBXW7, MLH1, MRE11A, MSH2, MSH6, NBN, NF1, NF2, NOTCH1, NOTCH2, NOTCH3, PALB2, PIK3R1, PMS2, POLE, PTCH1, PTEN, RAD50, RAD51, RAD51B, RAD51C, RAD51D, RB1, RNF43, SETD2, SLX4, SMARCA4, SMARCB1, STK11, TP53, TSC1, TSC2.  ***- Hotspot Genes (n=87); DNA***  AKT1, AKT2, AKT3, ALK, AR, ARAF, AXL, BRAF, BTK, CBL, CCND1, CDK4, CDK6, CHEK2, CSF1R, CTNNB1, DDR2, EGFR, ERBB2, ERBB3, ERBB4, ERCC2, ESR1, EZH2, FGFR1, FGFR2, FGFR3, FGFR4, FLT3, FOXL2, GATA2, GNA11, GNAQ, GNAS, H3F3A, HIST1H3B, HNF1A, HRAS, IDH1, IDH2, JAK1, JAK2, JAK3, KDR, KIT, KNSTRN, KRAS, MAGOH, MAP2K1, MAP2K2, MAP2K4, MAPK1, MAX, MDM4, MED12, MET, MTOR, MYC, MYCN, MYD88, NFE2L2, NRAS, NTRK1, NTRK2, NTRK3, PDGFRA, PDGFRB, PIK3CA, PIK3CB, PPP2R1A, PTPN11, RAC1, RAF1, RET, RHEB, RHOA, ROS1, SF3B1, SMAD4, SMO, SPOP, SRC, STAT3, TERT, TOP1, U2AF1, XPO1.  ***- Copy number variations (n=47); DNA***  AKT1, AKT2, AKT3, ALK, AR, AXL, BRAF, CCND1, CCND2, CCND3, CCNE1, CDK2, CDK4, CDK6, EGFR, ERBB2, ESR1, FGF19, FGF3, FGFR1, FGFR2, FGFR3, FGFR4, FLT3, IGF1R, KIT, KRAS, MDM2, MDM4, MET, MYC, MYCL, MYCN, NTRK1, NTRK2, NTRK3, PDGFRA, PDGFRB, PIK3CA, PIK3CB, PPARG, RICTOR, TERT.  ***- Gene Fusions (n=51); RNA***  ALK, AXL, BRAF, EGFR, ERBB2, ERG, ETV1, ETV4, ETV5, FGFR1, FGFR2, FGFR3, NTRK1, NTRK3, PDGFRA, PPARG, RAF1, RET, ROS1, AKT2, AR, BRCA1, BRCA2, CDKN2A, ERB84, ESR1, FGR, FLT3, JAK2, KRAS, MDM4, MET, MYB, MYBL1, NF1, NOTCH1, NOTCH4, NRG1, NTRK2, NUTM1, PDGFRB, PIK3CA, PRKACA, PRKACB, PTEN, RAD51B, RB1, RELA, RSPO2, RSPO3, TERT. |
| **Oncomine Childhood Cancer Research Assay** |
| ***- Completely Covered Genes (n=44); DNA***  APC, ARID1A, ARID1B, ATRX, CDKN2A, CDKN2B, CEBPA, CHD7, CRLF1, DDX3X, DICER1, EBF1, EED, FAS, GATA1, GATA3, GNA13, ID3, IKZF1, KDM6A, KMT2D, MYOD1, NF1, NF2, PHF6, PRPS1, PSMB5, PTCH1, PTEN, RB1, RUNX1, SMARCA4, SMARCB1, SOCS2, SUFU, SUZ12, TCF3, TET2, TP53, TSC1, TSC2, WHSC1, WT1, XIAP.  ***- Hotspot Gene (n=86); DNA***  ABL1, ABL2, ALK, ACVR1, AKT1, ASXL1, ASXL2, BRAF, CALR, CBL, CCND1, CCND3, CCR5, CDK4, CIC, CREBBP, CRLF2, CSF1R, CSF3R, CTNNB1, DAXX, DNMT3A, EGFR, EP300, ERBB2, ERBB3, ERBB4, ESR1, EZH2, FASLG, FBXW7, FGFR1, FGFR2, FGFR3, FLT3, GATA2, GNA11, GNAQ, H3F3A, HDAC9,  HIST1H3B, HRAS, IDH1, IDH2, IL7R, JAK1, JAK2, JAK3, KDM4C, KDR, KIT, KRAS, MAP2K1, MAP2K2, MET, MPL, MSH6, MTOR, MYC, MYCN, NCOR2, NOTCH1, NPM1, NRAS, NT5C2, PAX5, PDGFRA, PDGFRB, PIK3CA, PIK3R1, PPM1D, PTPN11, RAF1, RET, RHOA, SETBP1, SETD2, SH2B3, SH2D1A, SMO, STAT3, STAT5B, TERT, TPMT, USP7, ZMYM3.  ***- Copy Number Variations (n=28); DNA***  ABL2, ALK, BRAF, CCND1, CDK4, CDK6, EGFR, ERBB2, ERBB3, FGFR1, FGFR2, FGFR3, FGFR4, GLI1, GLI2, IGF1R, JAK1, JAK2, JAK3, KIT, KRAS, MDM2, MDM4, MET, MYC, MYCN, PDFRA, PIK3CA.  ***- Gene Fusions (n=91); RNA***  ABL1, ABL2, AFF3, ALK, BCL11B, BCOR, BCRF, BRAF, CAMTA1, CCND1, CIC, CREBBP, CRLF2, CSF1R, DUSP22, EGFR, ETV6, EWSR1, FGFR1, FGFR2, FGFR3, FLT3, FOSB, FUS, GLI1, GLIS2, HMGA2, JAK2, KAT6A, KMT2A, KMT2B, KMT2C, KMT2D, LMO2, MAML2, MAN2B1, MECOM, MEF2D, MET, MKL1, MLLT10, MN1, MYB, MYBL1,MYH11, MYH9, NCOA2, NCOR1, NOTCH1, NOTCH2, NOTCH4, NPM1, NR4A3, NTRK1, NTRK2, NTRK3, NUP214, NUP98, NUTM1, NUTM2B, PAX3, PAX5, PAX7, PDGFB, PDGFRA, PDGFRB, PLAG1, RAF1, RANBP17, RARA, RECK, RELA, RET, ROS1, RUNX1, SS18, SSBP2, STAG2, STAT6, TAL1, TCF3, TFE3, TP63, TSLP, TSPAN4, UBTF, USP6, WHSC1, YAP1, ZMYND11, ZNF384.  ***- Gene expression (n=9); RNA***  BCL2, BCL6, FGFR1, FGFR4, IGF1R, MET, MYC, MYCN, TOP2A. |

**Supplementary Table 3:** **Scores for classification of actionable alterations.**

| **Classification** | **Scores** |
| --- | --- |
| **OncoKB**  Memorial Sloan Kettering Cancer Center’s Oncology Knowledge Base (OncoKB) is a tumor mutation database that displays biological and oncogenic effects and the prognostic and predictive significance of somatic molecular alterations. Potential treatment implications are stratified by the level of evidence [1]. | - **Level 1:** (FDA)–recognized biomarkers that are predictive of response to an FDA-approved drug in a specific indication - **Level 2A:** standard care biomarkers that are predictive of response to an FDA-approved drug in a specific indication - **Level 2B:** FDA-approved biomarkers predictive of response to an FDA-approved drug detected in an off-label indication - **Level 3A:** biomarkers that are predictive of response to novel targeted agents that have shown promising results in clinical trials - **Level 3B:** biomarkers that are predictive of response to a drug in another indication, but neither biomarker nor drug is standard care - **Level 4:** biomarkers that are predictive of response to novel targeted agents on the basis of compelling biologic data |
| **ESCAT score**  ESMO Scale for Clinical Actionability of molecular Targets (ESCAT) is a collaborative project to propose a classification system for molecular aberrations based on the evidence available supporting their value as clinical targets [2]. | - **I-A:** Prospective, randomised clinical trials show the alteration-drug match in a specific tumor type results in a clinically meaningful improvement of a survival end point - **I-B:** Prospective, non-randomised clinical trials show that the alteration-drug match in a specific tumor type, results in clinically meaningful benefit as defined by ESMO MCBS 1.1 - **I-C**: Clinical trials across tumour types or basket clinical trials show clinical benefit associated with the alteration-drug match, with similar benefit observed across tumour types - **II-A:** Retrospective studies show patients with the specific alteration in a specific tumour   type experience clinically meaningful benefit with matched drug compared with alteration-negative patients   - **II-B:** Prospective clinical trial(s) show the alteration-drug match in a specific tumour type results in increased responsiveness when treated with a matched drug, however, no data currently available on survival end points - **III-A:** Clinical benefit demonstrated in patients with the specific alteration (as tiers I and II   above) but in a different tumour type. Limited/absence of clinical evidence available for  the patient-specific cancer type or broadly across cancer types   - **III-B:** An alteration that has a similar predicted functional impact as an already studied tier I abnormality in the same gene or pathway, but does not have associated supportive clinical data - **IV-A:** Evidence that the alteration or a functionally similar alteration influences drug   sensitivity in preclinical in vitro or in vivo models   - **IV-B:** Actionability predicted in silico - **V:** Prospective studies show that targeted therapy is associated with objective responses, but this does not lead to improved outcome |
| **NCT/DKTK classification**  A classification to assess molecular biomarkers beyond approved “standard of care” targets [3]. | - **m1A:** The predictive value of the biomarker or clinical effectiveness of the corresponding drug in a molecularly stratified cohort was demonstrated in a prospective study or meta-analysis in the same tumor type - **m1B:** The predictive value of the biomarker or clinical effectiveness of the drug in a molecularly stratified cohort was demonstrated in a retrospective cohort or case-control study in the same tumor type. - **m1C:** A case study or single unusual responder indicates that the biomarker is associated with response to the corresponding drug in the same tumor type - **m2A:** The predictive value of the biomarker or clinical effectiveness of the corresponding drug in a molecularly stratified cohort was demonstrated in a prospective study or meta-analysis in a different tumor type - **m2B:** The predictive value of the biomarker or clinical effectiveness of the drug in a molecularly stratified cohort was demonstrated in a retrospective cohort or case-control study in a different tumor type - **m2C:** The predictive value of the biomarker or clinical effectiveness of the drug in a molecularly stratified cohort was demonstrated in a retrospective cohort or case-control study in a different tumor type - **m3:** Preclinical data demonstrate that the biomarker predicts response to a specific drug, supported by a scientific rationale - **m4:** A biological rationale exists that associates the biomarker with altered activity of cellular pathways/processes or drug sensitivity without direct clinical or preclinical evidence for a response to the drug |

**Supplementary Table 4:** **List of actionable mutations in the panel gene sequencing cohort.** Actionability of specific mutations was assessed based on the OncoKB database (OnkoKB score), ESMO treatment guidelines (ESCAT score) and recommendations of the local molecular tumor board (NCT/DKTK score).

| **Gene** | **Mutation** | **Cancer entity** | **Suggested therapy** | **OncoKB**  **score** | **ESCAT score** | **NCT/DKTK score** |
| --- | --- | --- | --- | --- | --- | --- |
| AKT1 | p.E17K | CRC/CCC | AZD5363 | 3B | - | ND |
| AKT2 | amplification | CRC | Capivasertib, Ipatasertib | - | - | m4 (Capivasertib, Ipatasertib) |
| ARID1A | p.D647Efs*28 | CCC | PLX2853, Tazemetostat | 4 | - | ND |
| ARID1A | p.G1293Efs*2 | CUP | PLX2853, Tazemetostat | 4 | - | ND |
| ARID1A | p.I1693Nfs*2 | HCC | PLX2853, Tazemetostat | 4 | - | ND |
| ARID1A | p.P121Afs*108 | CCC | PLX2853, Tazemetostat | 4 | - | ND |
| ARID1A | p.Q1148* | CCC | PLX2853, Tazemetostat,  Nivolumab + Ipilimumab | 4 | - | m1A (Nivolumab + Ipilimumab) |
| ARID1A | p.Q588* | HCC | PLX2853, Tazemetostat | 4 | - | ND |
| ARID1A | p.T1514Rfs*13 | CCC | PLX2853, Tazemetostat,  Capivasertib + Olaparib | 4 | - | m1c (Capivasertib + Olaparib) |
| ATM | p.K24Rfs*10 | CRC | Talazoparib + Enzalutamide; Olaparib | 3B | - | ND |
| ATM | p.R3008C | CRC | Talazoparib + Enzalutamide; Olaparib | 3B | - | ND |
| BAP1 | p.R59Q | CCC | Olaparib | - | - | m4 |
| BRAF | p.V600E | CRC | Encorafenib + Cetuximab | 1 | I-A | ND |
| BRCA1 | p.E1161Ffs*3 | PDAC | Olaparib; Olaparib + Bevacizumab; Niraparib; Rucaparib; Olaparib + Abiraterone + Prednisone; Olaparib + Abiraterone + Prednisolone, Talazoparib + Enzalutamide | 3A (Olaparib) | I-A  (Olaparib) | m1A  (Olaparib) |
| BRCA2 | p.K3326* | CCC | Olaparib | - | III-A | NA |
| BRCA2 | p.S497L | PDAC | Olarparib | - | I-A | m1A |
| BRCA2 | p.Y2726Vfs*5 | PDAC | Olaparib; Olaparib + Bevacizumab; Niraparib; Rucaparib; Olaparib + Abiraterone + Prednisone; Olaparib + Abiraterone + Prednisolone, Talazoparib + Enzalutamide | 3B | I-A (Olaparib) | ND |
| CDKN2A | p.D108N | HCC | Palbociclib, Ribociclib, Abemaciclib | 4 | - | ND |
| CDKN2A | p.L32R | Ampullary cancer | Palbociclib, Ribociclib, Abemaciclib | 4 | - | ND |
| EML4 (6) – ALK (20) | translocation | CRC | Alectinib, Brigatinib, Ceritinib, Crizotinib, Lorlatinib, Entrectinib | 3B | III-A (Entrectinib) | ND |
| ERBB2 | amplification | PDAC | Trastuzumab + Pertuzumab, Lapatinib + Trastuzumab, Pembrolizumab + Trastuzumab + Chemotherapy | 3B | - | ND |
| ERBB2 | p.R678Q | CRC | Ado-Trastuzumab Emtansine, Trastuzumab Deruxtecan, Neratinib, Trastuzumab + Pertuzumab + Docetaxel | 3B | - | ND |
| ERBB2 | p.T733I | EGJ | Ado-Trastuzumab Emtansine, Trastuzumab Deruxtecan, Neratinib, Trastuzumab + Pertuzumab + Docetaxel | 3B | - | m2A (Trastuzumab-Deruxtecan) |
| ERBB2 | p.V842I | PDAC | Ado-Trastuzumab Emtansine, Trastuzumab Deruxtecan, Neratinib, Trastuzumab + Pertuzumab + Docetaxel | 3B | - | m2A (Neratinib, Trastuzumab Emtansine)  m2B (Afatinib) |
| ERBB3 | p.G284R | CCC | Afatinib; Trastuzumab/ Lapatinib | - | - | m2C  (Afatinib; Trastuzumab/ Lapatinib) |
| ERBB3 | p.V104L | CCC | Afatinib | - | - | m2A |
| FANCA | p.A1219Gfs*59 | CRC | Talazoparib + Enzalutamide | 3B | - | ND |
| FANCA | p.R435H | EGJ | Talazoparib + Enzalutamide | 3B | - | ND |
| FGFR1 | amplification | CRC | Erdafitinib | 3B | - | ND |
| GOPC (4) – ROS1 (36) | gene fusion | CRC | Crizotinib, Entrectinib, Repotrectinib, Ceritinib, Lorlatinib | 3B | - | ND |
| HRAS | p.Q61L | CCC | Tipifarnib | 3B | - | ND |
| IDH1 | p.R132C | CCC | Ivosidenib | 1 | I-A | ND |
| IDH1 | p.R132C | CRC | Ivosidenib | 3B | - | ND |
| IDH2 | p.R172K | CCC | Enasidenib | 3B | III-A | ND |
| KIT | p.M552T | Gastric cancer | Imatinib | - | - | m4 |
| KRAS | p.A146T | CRC | Trametinib, Cobimetinib, Binimetinib | 4 | - | ND |
| KRAS | p.G12A | CRC | Trametinib, Cobimetinib, Binimetinib; RMC-6236 | 4 | - | ND |
| KRAS | p.G12C | PDAC | Adagrasib, Adagrasib + Cetuximab; Sotorasib | 3B | - | m2A  (Sotorasib) |
| KRAS | p.G12D | CRC; PDAC | MRTX-1133; ASP308; Trametinib, Cobimetinib, Binimetinib; RMC-6236 | 4 | - | ND |
| KRAS | p.G12R | PDAC | Trametinib, Cobimetinib, Binimetinib; RMC-6236 | 4 | - | ND |
| KRAS | p.G12S | CRC | Trametinib, Cobimetinib, Binimetinib; RMC-6236 | 4 | - | ND |
| KRAS | p.G12V | CRC; CCC; Gastric cancer; PDAC | Trametinib, Cobimetinib, Binimetinib; RMC-6236 | 4 | - | ND |
| KRAS | p.G13D | CRC | Trametinib, Cobimetinib, Binimetinib | 4 | - | ND |
| KRAS | p.K117N | CRC | Trametinib, Cobimetinib, Binimetinib | 4 | - | ND |
| KRAS | p.Q61H | PDAC | Trametinib, Cobimetinib, Binimetinib | 4 | - | ND |
| MET | amplification | HCC | Tepotinib | 3B |  | m1A |
| MLH1 | p.E671Dfs*112 | Duodenal cancer | Talazoparib + Enzalutamide | 3B | - | m2A |
| MLH1 | p.R100* | Duodenal cancer | Talazoparib + Enzalutamide | 3B | - | m2A |
| MTOR | p.V2006L | CRC | Everolimus Temsirolimus | 4 | - | ND |
| MYC | amplification | EGJ | IDH2 Inhibitor | - | - | m4 |
| NBN | p.K219N*16 | EGJ | Olaparib | - | - | m2C |
| NF1 | p.D1194* | CRC | Trametinib, Cobimetinib | 4 | - | ND |
| NF1 | p.I679Dfs*21 | CRC | Trametinib, Cobimetinib | 4 | - | ND |
| NRAS | p.G12D | CCC | Binimetinib | 3B | - | ND |
| NRAS | p.Q61K | CCC | Binimetinib | 3B | - | ND |
| NRAS | p.Q61L | CRC | Binimetinib | 3B | - | ND |
| NRAS | p.Q61R | CRC | Binimetinib | 3B | - | ND |
| PALB2 | p.S804Cfs*10 | Duodenal cancer | Olaparib; Talazoparib + Enzalutamide; Rucaparib | 3B | - | m2A (PARP inhibitor) |
| PIK3CA | p.E542K | CRC; EGJ | Alpelisib; RLY-2608 | 3B | - | ND |
| PIK3CA | p.H1047L | CRC | Alpelisib; RLY-2609 | 3B | - | ND |
| PIK3CA | p.H1047R | CRC; CCC | Alpelisib; RLY-2610 | 3B | - | ND |
| PIK3CA | p.H1047Y | CRC | Alpelisib; RLY-2611 | 3B | - | ND |
| PIK3CA | p.K111E | CRC | Alpelisib; RLY-2612 | 3B | - | ND |
| PIK3CA | p.T1052K | CRC | Alpelisib; RLY-2613 | 3B | - | m2C (Alpelisib) |
| PTCH1 | p.Q1300Hfs*25 | CRC | Sonidegib, Vismodegib | 3B | - | ND |
| RB1 | p.Q257* | EGJ | Palbociclib | - | - | m3 |
| SMARCA4 | p.R397* | EGJ | CDK4/6 Inhibitor + Immune Checkpoint Inhibitor | - | - | m2C |
| SMARCA4 | p.R905Afs*5 | CCC | CDK4/6 Inhibitor + Immune Checkpoint Inhibitor | - | - | m2C |
| TP53 | p.Y220C | PDAC | PC14586 | 3A | - | ND |
| TSC2 | p.Q166* | CRC | ABI-009, Everolimus | 3B | - | ND |

*Abbreviations: CCC* Cholangiocellular carcinoma, *CRC* Colorectal cancer, *CUP* Cancer of unknown primary, *EGJ* Esophagogastric junction adenocarcinoma, *HCC* Hepatocellular carcinoma, *PDAC* pancreatic ductal adenocarcinoma, *ND* not discussed (cases were not presented in local molecular tumor board).

**Additional references**

1. Chakravarty D, Gao J, Phillips S, Kundra R, Zhang H, Wang J, et al. OncoKB: A Precision Oncology Knowledge Base. JCO Precis Oncol. 2017;2017:1–16.

2. Mateo J, Chakravarty D, Dienstmann R, Jezdic S, Gonzalez-Perez A, Lopez-Bigas N, et al. A framework to rank genomic alterations as targets for cancer precision medicine: the ESMO Scale for Clinical Actionability of molecular Targets (ESCAT). Ann Oncol. 2018;29:1895–902.

3. Lier A, Penzel R, Heining C, Horak P, Fröhlich M, Uhrig S, et al. Validating Comprehensive Next-Generation Sequencing Results for Precision Oncology: The NCT/DKTK Molecularly Aided Stratification for Tumor Eradication Research Experience. JCO Precis Oncol. 2018;2:1–13.
